# The treatment of aneurysmal bone cysts

**DOI:** 10.1097/MOP.0000000000001205

**Published:** 2022-11-21

**Authors:** Thomas P.G. van Geloven, Michiel A.J. van de Sande, Lizz van der Heijden

**Affiliations:** Department of Orthopaedics, Leiden University Medical Center, Leiden, The Netherlands

**Keywords:** aneurysmal bone cysts, curettage, denosumab, sclerotherapy, surgery

## Abstract

Aneurysmal bone cysts are rare, locally aggressive bone tumors. Optimal treatment of ABCs is still matter of debate as therapies including sclerotherapy, selective arterial embolization and systemic treatment with denosumab are increasingly utilized, in addition to or instead of traditional curettage. The purpose of this review is to discuss current concepts and difficulties in diagnosing and treating primary ABCs, based on latest available literature.

**Recent findings:**

In diagnostics, multiple new fusion partners of USP-6 have been described on next-generation sequencing specifically for primary ABCs. In a recent systematic review, failure rates of percutaneous injections and surgery were comparable. In a literature review, the use of denosumab seemed effective but resulted in multiple cases of severe hypercalcemia in children.

**Summary:**

Accurately diagnosing primary ABC is crucial for treatment decisions. Curettage remains a valid treatment option, especially with adjuvant burring, autogenous bone grafting and phenolization. Percutaneous sclerotherapy represents a solid alternative to surgery, with polidocanol showing good results in larger studies. Systematic therapy with denosumab exhibits favorable results but should be reserved in the pediatric population for unresectable lesions, as it may result in severe hypercalcemia in children. When selecting a treatment option, localization, stability and safety should be considered.

## INTRODUCTION

Aneurysmal bone cysts (ABC) are rare, locally aggressive bone tumors of cystic and osteolytic nature [1,2^▪^]. Seventy-five to 90% of ABCs are diagnosed in the first two decades of life [3], with the mean age at 13 years [4]. ABCs often present with complaints of pain, a palpable mass, pathological fracture or with neurological symptoms in case of spinal ABC [1,5^▪▪^].

Conventional radiographs show expansile, well defined osteolytic lesions with a soap bubble appearance and fluid-like radio-opacity [2^▪^,^6^,^7^]. However, x-rays often fail to determine a definitive diagnosis [3]. The gold standard for diagnosing ABC is MRI, combined with biopsy [2^▪^,^3^,^4^,8^▪^]. On MRI, cystic lesions can be seen, often with fluid–fluid levels, surrounded by fibrous septae and borders with hypointense properties [9^▪^]. Imaging of these borders and septae can be enhanced with intravenous contrast agent [10^▪^].

Microscopically, ABC have spindled fibroblast proliferations, with osteoclast-like giant cells and stromal mononuclear cells [11,12]. Genetically, primary ABC has rearrangements of *USP6* and/or *CDH11* genes in 70% of cases, differentiating them from formerly called secondary ABCs (ABC-like area would be more correct) [1], which are regarded as morphological mimics with a common ABC-like endpoint lacking these specific mutations [1,11]. This increases the importance of proper diagnostics, as both benign and malignant bone tumors can have ABC-like areas. Therefore, included in the differential diagnosis of these cystic lesions in children and adolescents are: giant cell tumor of bone (GCTB), osteoblastoma, chondroblastoma, chondromyxoid fibroma, simple bone cysts (SBC), fibrous dysplasia, nonossifying fibroma (NOF) and (telangiectatic) osteosarcoma [1,3]. Especially telangiectatic osteosarcoma is important in the differential diagnosis, as imaging characteristics are very similar to ABC, and as the only malignancy in the differential diagnosis, it should not be missed [1,13].

Preferred treatment of ABC has always been up for debate, as no single treatment modality has universal preference. This article will discuss the current concepts and difficulties in the diagnostics and treatment of ABCs based on the latest available evidence. 

**Box 1:**
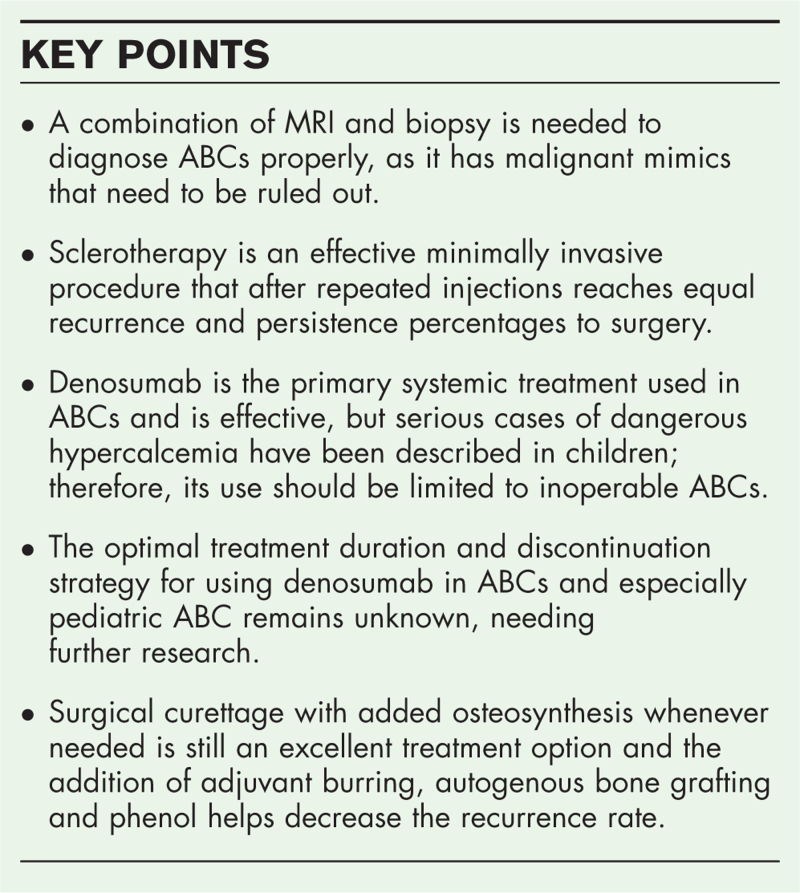
no caption available

## RADIOLOGY

A useful clinically based description of ABCs on MRI was recently written by Josip *et al.*[9^▪^]. Circumferential enhancement from intravenous contrast was observed in all ABCs but in SBCs as well. The presence or absence of internal septations or hypointense outer rim could likewise correlate to both ABC and SBC, reinstating the importance of additional histology.

The study of Cui *et al.*[14^▪^] looked into differentiating primary ABC and GCTB with ABC-like areas. Apart from clinical differences in population groups like older age in GCTB (19.3 vs. 12.7 years), they found subchondral bone involvement (92.3 vs. 30.8%) and deep lobulation (38.5 vs. 0%) to arise more likely in GCTBs. Surrounding blood vessels adjacent to the lesion were solely observed in ABCs (0 vs. 46%).

## PATHOLOGY

The differentiation of all giant cell-rich bone tumors has made significant steps over the past years [15^▪▪^]. Using multiple core biopsies with 16 or 18 gauge needles, it is possible to use histology, touch preparations, immunohistochemistry (IHC) and fluorescence in-situ hybridization (FISH) for diagnosis. histology showed hemosiderin depositions, foamy macrophages and woven bone formation in addition to the classic solid and cystic areas filled with blood and surrounded by fibrous septa. Touch preparation slides of ABC lesions were blood-rich and hypocellular, with multinucleated osteoclast-like giant cells, bland spindle cells and lymphocytes, macrophages and rare neutrophils. On IHC, no expression of H3G34W (normally related to GCTB), H3K36M (chondroblastoma), or SATB2 (osteosarcoma with giant cells) was seen. On FISH, USP6 rearrangements were seen exclusively in primary ABCs.

Increased use of next generation sequencing (NGS) for diagnosing ABCs has resulted in continuous uncovering of new USP-6 fusion partners in ABCs and improved bone cyst distinguishing [16^▪^,^17^,^18^]. Another study confirmed this and also examines nodular fasciitis and myositis ossificans, benign tumors with similar USP-6 rearrangements but different fusion partners [19^▪^].

After surgery, additional histological assessment is often conducted to confirm the diagnosis. This may be challenging in cases where neoadjuvant denosumab has been given as described by Hung *et al.*[20] showing altered histology, with elongated, thin curvilinear and anastomosing neoplastic woven bone strands with empty lacunae. Blue bone was observed in this configuration, as was endochondral ossification of cartilage. The appearance was similar to fibrous dysplasia or parosteal osteosarcoma. In these cases, histological assessment after resection can be challenging. However, with the clinical history of denosumab use, misdiagnoses can be avoided.

In the most recent WHO classification, the term secondary ABC was abandoned in favor of ABC-like area, as the latter encourages maltreatment of the actual underlying neoplasm.

## TREATMENT MODALITIES

**FIGURE 1:**
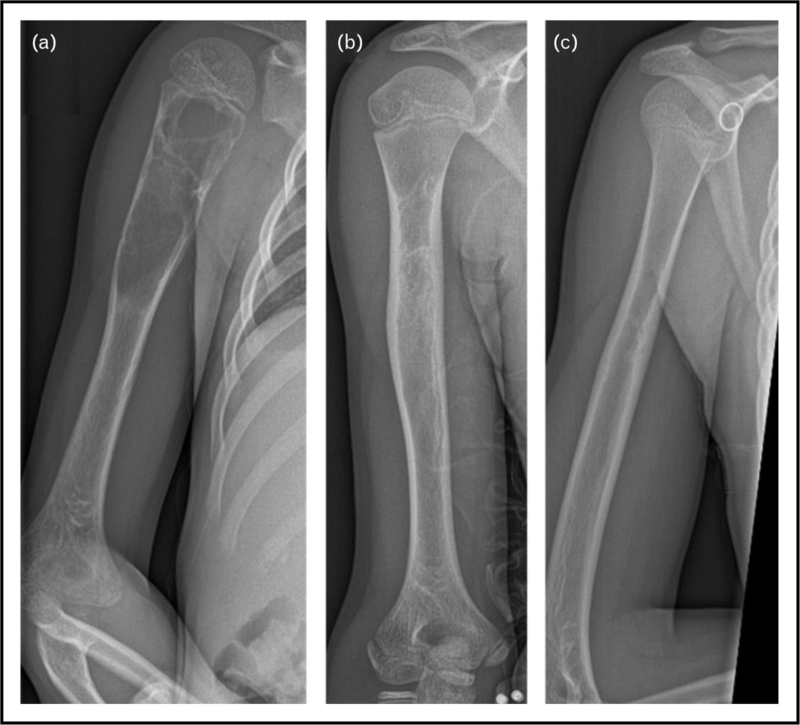
(a) An aneurysmal bone cyst in the right proximal humerus with well defined septa creating a soap-bubble apearance treated with ethoxysclerol. (b) The lesion has moved further from the epiphyseal plate because of growth. After first ethoxysclerol injection, the intracameral septa had largely disappeared and the previously well defined borders became vaguer. Patient received a total of three injections of ethoxysclerol. (c) At the end of follow-up, consolidation of the cyst and complete remodulation of the humeral shaft was observed.

**FIGURE 2:**
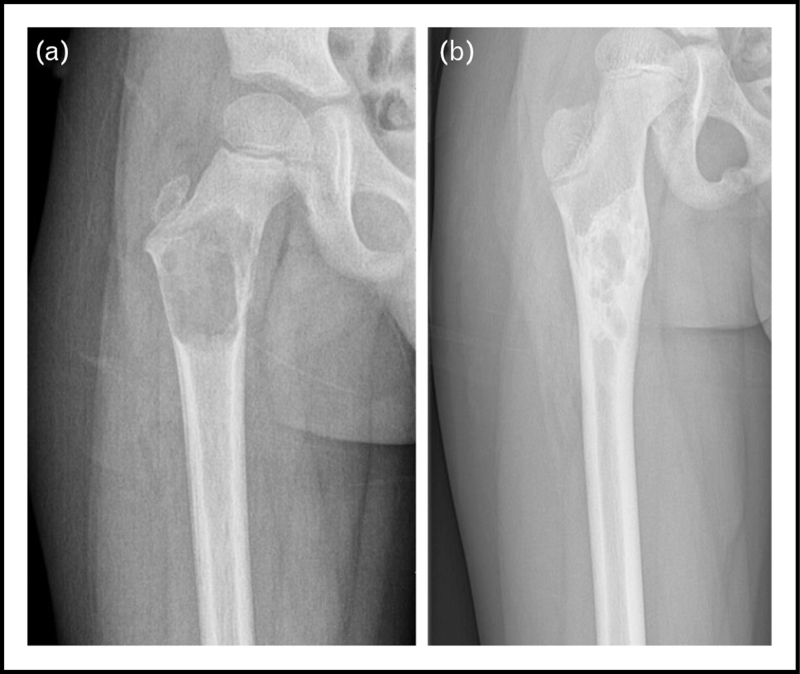
(a) An aneurysmal bone cyst in the right proximal femur, exhibiting fluid-like radio-opacity and cortical destruction. (b) After curettage with bone grafting filling and remodulation was seen with increased cortical stability at the end of follow-up.

There is a wide array of treatment options currently in use, with percutaneous sclerotherapy (Fig. 1) and surgical procedures (Fig. 2) as two of the most frequently utilized. With no one modality having been proven superior to the others, this remains a topic of much scholarly debate. We have highlighted the developments of the past period.

## PERCUTANEOUS PROCEDURES

Over the last years, a growing interest in percutaneous treatment for ABCs has been seen, ranging from percutaneous curettage techniques [21] to the wide array of injectable agents and embolizations [22]. The less invasive nature of percutaneous procedures combined with faster return to full weight bearing as described by van Geloven *et al.* and the possibility for use in difficult to reach locations, explain its increasing popularity [23]. Bavan *et al.* reported in their systematic review of 28 articles that injection therapies and surgical modalities have similar failure rates (14.7 compared with 14.4%) [24]. They pointed out, however, with ABCs mainly affecting the pediatric population, the considerable burden of multiple general anesthetics for serial injection therapies should be considered.

### Sclerotherapy

Many sclerotherapies have been described with different sclerosants and application techniques. Some like sodium tetradecyl sulfate are injected with air to create a foam [25^▪^], while others like polidocanol are injected as liquids [5^▪▪^]. Sometimes combinations with embolizing substances are administered, aiming for increasing safety [26^▪▪^].

A meta-analysis by Cruz *et al.* including 10 studies (294 patients) utilizing either polidocanol, Ethibloc, Doxycycline, calcitonin and steroid or calcium sulfate, reported recurrence-free survival of 94% at final follow-up (mean = 41.1, range = 23–58 months) [27]. However, 68 of 294 patients had complications including injection site induration, skin necrosis and fractures.

Radiologically, Bih *et al.*[10^▪^] described decreased cystic areas and improved cortical integrity in over half their cases, on average 5.9 and 10.6 months after initial sclerotherapy. However, paired with increased edema in 19% of cases and presence of fibrosis in 53%.

At present, no single sclerotherapy modality has been proven superior to the others, with agent choice being dependent on individual center preferences. However, usage of Ethibloc has rapidly declined because of the possibility of serious complications, and H_2_O_2_ has recently been described to cause severe acute lung injury [28]. As many safer alternatives exist, the use of these substances should be reconsidered.

### Doxycycline

Originally an antibiotic, in bone tumors, doxycycline demonstrates antitumoral properties and functions as a sclerosing agent. Two hundred milligrams of doxycycline, with 5 ml albumin 25% and 10 ml of air creates foam as first described by Shiels *et al.*[29^▪▪^,^31^]. This agent is not widely adapted but two articles appeared recently. Arleo *et al.*[32^▪^] reported treatment with doxycycline alone or combining cryotherapy with recurrence free healing in 13/15 cases [30]. Wong *et al.*[33^▪^] reported 14 patients who received doxycyline in cervical spine ABCs, achieving a success rate of 86% [31]. Reported complications were transient paralysis and transient circulation strokes for spinal lesions [30].

### Polidocanol

The large retrospective study of Jasper *et al.*[5^▪▪^] describes the success rates and risk factors for treatment failure and complications of polidocanol injections in 70 patients with 83% healing rate with polidocanol alone and 75% 5-year recurrence-free survival. Lower leg involvement had a three-fold increased risk for failure compared with other localizations. Kumar *et al.*[32^▪^] described significant and swift pain relief and cyst healing with repeated polidocanol injections.

Deventer *et al.*[49] had disease persistence in 90.6% of 32 cases, after first injection, eventually requiring a mean of 5.7 injections, whereas Rai *et al.* reported all 43 patients to have fully resolved lesions after 2 years with 3–4 injections [33^▪^,^34^]. Multiple injections are common, with a mean number of injections varying between 1.8 and 5.7 [5^▪▪^,32^▪^,33^▪^,^34^]. Complications observed are: skin induration at injection site, skin hypopigmentation [32^▪^,^34^] and rarely anaphylactic shock [5^▪▪^].

Puthoor *et al.*[35^▪^] compared polidocanol sclerotherapy to curettage with bone grafting and found similar clinical and radiological outcomes but less complications, better functional scores and lower costs for sclerotherapy.

### Denosumab

Denosumab is a monoclonal anti-RANK-L antibody used to treat bone loss because of increased osteoclast activity. It prevents osteoclast differentiation and activity by binding RANK ligand. The use of denosumab in particular has been shown effective as rescue therapy for controlling ABCs [2^▪^]. However, a growing number of articles were published, like Deodati *et al.* and Del Sindaco *et al.* warning for severe adverse events after discontinuation in children [36^▪^,37^▪^]. Deodati *et al.* report a 10-year-old boy with ABC, who after 10 months of high-dose denosumab treatment developed a hypercalcemia with nephrocalcinosis. Three doses of bisphosphonates were administered, leading to serum calcium stabilization. Del Sindaco *et al.* report an 8-year-old boy with a nonresectable ABC of cervical vertebrae (C4-C7) with vascular involvement, treated with denosumab. He was treated with five doses with 4-month intervals?, after which optimal clinical response was seen and denosumab was stopped. Nine months after his last dose, recurrence occurred and denosumab was started again for 2.5 years, after which the tumor was described as stable and calcified. However, during treatment period, three episodes of hypercalcemia occurred for which the patient received 6-monthly zoledronic acid infusions to stabilize the values. He also developed genu valgum, eventually requiring bilateral epiphysiodesis.

Both studies emphasize the importance of establishing optimal treatment duration, having a gradual discontinuation strategy and long-term monitoring for growth (deformities), serum calcium and mineral homeostasis. Earlier studies suggested the use of bisphosphonates as a preventive strategy [38^▪^], and these studies confirm the usefulness [36^▪^,37^▪^]. However, knowledge regarding consequences for the growing skeleton and strong evidence to the effectiveness of bisphosphonates in ABCs are lacking.

A review by Maximen *et al.* divided the study group of 45 patients in below 15 (pediatric) and above 15 years old (adult) [39^▪^]. The pediatric population experienced significantly fewer recurrences (1 vs. 7). However, 6 of 21 children treated with denosumab (28.6%) developed symptomatic hypercalcemia of whom 5 needed admission to the ICU.

This high chance of serious complications allows denosumab to be reserved as rescue therapy in unresectable ABCs or in those with multiple recurrences and no alternative.

### Percutaneous curettage

Alisi *et al.* discussed a percutaneous technique for curettage through a 1 cm incision [21]. With two size-16 jamshidi needles, all intracystic fluid was drained, after which surrounding metaphyseal bone was curetted with an angled curette or bent tip for an 5 mm Steinmann pin/5 mm drill bit, followed by cancellous bone cyst filling. Locations were proximal humerus, radius, distal and proximal femur and tibia. Re-procedure was performed in one of nine patients. All cases had full healing without complications.

### Selective arterial embolization

Selective arterial embolization as primary treatment has been described for cases in hard to reach locations like the pubic bone [40], sacrum and spine [41^▪^]. In the Cruz *et al.* study, SAE with either N-2-butyl-cyanoacrylate or PVA particles showed an 18% recurrence rate and a complication rate of only 3% including skin necrosis, sciatic nerve paresthesia and artery pseudoaneurysms [27].

Currently, embolization is mostly used combined with sclerotherapy in hard-to-reach places [26^▪▪^], or as preoperative adjunct to surgery [41^▪^]. Although these studies mostly consist of case reports and SAE effectiveness lacks hard evidence, it is still a promising treatment modality deserving extensive research.

## SURGICAL PROCEDURES

The single-centered retrospective study of Döring *et al.* on surgical treatment of ABCs covers some of the latest insights in using curettage with or without adjuvants and/or filling [42]. They reported an overall recurrence rate of 28 of 90 patients (31%), and a 1, 2 and 5 years recurrence-free survival of 83, 77 and 66%, respectively. In their subgroup analyses, age below 10 showed lower RFS, while adjuvant burring and autogenous bone grafting usage resulted in higher RFS, with a trend in favor of using phenol (*P* = 0.051). Filling with allogenous bone grafts, bone cement or no filling did not affect RFS.

Bombardier *et al.* evaluated necrosis depth of different local adjuvants (bipolair hemostatic sealer 0.9 mm, phenol 0.3 mm, liquid nitrogen 2.5 mm, argon beam 2.5 mm, PMMA 0.8 mm) [43]. However, they mentioned that adequate necrosis depth and the point where increased complications occur are both unknown and await further studies.

Tomaszewski *et al.*[8^▪^] describes treatment of seven proximal femoral ABCs (six of whom presented with pathological fracture) using intralesional curettage and plate osteosynthesis. One patient needed re-procedure after recurrence. No re-fractures arose after this rigorous approach; however, one patient developed deep wound infection, which resolved after implant removal, wound debridement and antibiotics.

## OSTEOSYNTHESES

Treatment of ABCs in weightbearing bones sometimes requires osteosyntheses to secure or re-establish bone stability. External fixators have been described in case reports as effective after proximal femur fractures. Alongside this fixation, curettage with strut grafting and/or bone graft filling can be considered to treat the initial lesion [44^▪▪^]. Some cases in weightbearing bones may eventually necessitate arthroplasties because of instability, for example, with a collapsed femoral head [23,45^▪^].

The usefulness of computer model assistance and 3D printing for improved remodeling and osteosynthesis has been demonstrated by Luo *et al.*[46].

## TREATING ANEURYSMAL BONE CYSTS IN RARE LOCATIONS

A large amount of case series into rare locations of ABCs have been published recently [47,48^▪^]. Localization of ABC in the hands and feet had significantly less recurrences compared with other locations in Döring *et al.*[42]. Less aggressive treatment might be considered in these loci.

Spinal lesions in general have received a substantial amount of interest recently, with multiple studies showing effectiveness of sclerotherapy in this vulnerable anatomical location [25^▪^]. In their cervical spine ABC series, Wong *et al.* reported one treatment-related complication after doxycycline injection in a C2 ABC leading to sclerotherapy triggered spasm of the right posterior inferior cerebellar artery, resulting in distribution area infarction [31]. Contrastingly, Palmisciano *et al.* describe a large population of 220 spinal ABCs and ABC-like areas mostly treated with curettage or laminectomy/corpectomy, with as main complication 4 deaths, 3 of which were labeled secondary ABCs [22].

In pelvic and sacral ABCs, because of fragility of surrounding soft tissue structures, a growing interest for percutaneous procedures has been justified by reports of successful treatment with polidocanol, SAE or combination of the two [40,48^▪^]. However, for some larger lesions, surgical curettage remains the preferred option.

The evidence on treatment of head and neck ABCs is thin, because of the rarity of manifestation. A recent review by Richardson *et al.* described the mandible (37.1%), sinus (14.3%) and cranium (11.4%) as the most common sites [50]. Surgical excision was performed in 94.1% of cases but they also reported cases with chemotherapy alone, radiotherapy with surgery or all three combined. The reported recurrence/persistence rate of 5.6% was low compared with rates reported in the long bones [2^▪^]. Chemotherapy or radiation therapy is, however, in our opinion exorbitant for treating local benign lesions. Smith *et al.* confirmed frequent surgical excision (97%) in the cranium and found orbital involvement on imaging (hazard ratio = 3.26) and proptosis (hazard ratio = 3.73) as prognostic factors for recurrence or progression [51^▪^,^52^].

## CONCLUSION

This review highlighted the latest evidence and discussed current concepts and difficulties in the treatment of ABCs.

Diagnostics are the first and essential step in treating ABCs and a combination of MRI and biopsy is needed to differentiate from a variety of mimics. The addition of NGS in diagnostics allows more certainty when USP6 rearrangements are found. When not found, H3G34W, H3k36M and SATB2 can be examined to identify GCTB, chondroblastoma and osteosarcoma, respectively.

Sclerotherapy provides good results, but often requires multiple injections for optimal effect. However, in selected cases, it is an excellent treatment option as it is less invasive and cost effective.

Denosumab is the primary systemic treatment for ABCs and recent studies describe its effectiveness. However, in pediatric patients, severe hypercalcemia is a frequent complication (28.6%).

Selective arterial embolization shows promising results in hard to operate ABCs, but further research is needed.

Surgical curettage with adjuvants like high-speed burring, phenol and autogenous bone grafting still shows good results and can safely be applied to larger, aggressive defects. The use of (additional) osteosyntheses may be necessary in weight-bearing locations or in cases with fractures.

Treatment choice is dependent on localization, size, impending or actual pathological fractures and symptoms, as well as surrounding tissues. For each lesion, a personalized balance should be made between invasiveness, recurrence risk and possible complications.

## Acknowledgements

*None.*

### Financial support and sponsorship

*None.*

### Conflicts of interest

*There are no conflicts of interest.*

## REFERENCES AND RECOMMENDED READING

Papers of particular interest, published within the annual period of review, have been highlighted as:

▪ of special interest
▪▪ of outstanding interest
